# Review of Polymeric Materials in 4D Printing Biomedical Applications

**DOI:** 10.3390/polym11111864

**Published:** 2019-11-12

**Authors:** Ming-You Shie, Yu-Fang Shen, Suryani Dyah Astuti, Alvin Kai-Xing Lee, Shu-Hsien Lin, Ni Luh Bella Dwijaksara, Yi-Wen Chen

**Affiliations:** 1 School of Dentistry, China Medical University, Taichung City 404, Taiwan; eric@mail.cmu.edu.tw; 2 3D Printing Medical Research Center, China Medical University Hospital, Taichung City 404, Taiwan; Leekaixingalvin@gmail.com (A.K.-X.L.); nehemiah555@gmail.com (S.-H.L.); 3 Department of Bioinformatics and Medical Engineering, Asia University, Taichung City 413, Taiwan; cherryuf@gmail.com (Y.-F.S.); bellawindufebriyanti@gmail.com (N.L.B.D.); 4 3D Printing Medical Research Institute, Asia University, Taichung City 413, Taiwan; 5 Biomedical Engineering Study Program, Department of Physic, Faculty of Science and Technology, Univerisitas Airlangga, Surabaya 61115, Indonesia; suryanidyah@fst.unair.ac.id; 6 School of Medicine, China Medical University, Taichung City 404, Taiwan; 7 Graduate Institute of Biomedical Sciences, China Medical University, Taichung City 404, Taiwan

**Keywords:** 4D printing, additive manufacturing, shape memory material, smart materials, shape memory polymer

## Abstract

The purpose of 4D printing is to embed a product design into a deformable smart material using a traditional 3D printer. The 3D printed object can be assembled or transformed into intended designs by applying certain conditions or forms of stimulation such as temperature, pressure, humidity, pH, wind, or light. Simply put, 4D printing is a continuum of 3D printing technology that is now able to print objects which change over time. In previous studies, many smart materials were shown to have 4D printing characteristics. In this paper, we specifically review the current application, respective activation methods, characteristics, and future prospects of various polymeric materials in 4D printing, which are expected to contribute to the development of 4D printing polymeric materials and technology.

## 1. Introduction

### 1.1. Definition

Additive manufacturing (AM) technology, also known as 3D printing, had been around for decades and it has allowed users to fabricate custom 3D objects using computer software and computer aided design (CAD) [1]. In recent years, this technology has had huge impacts on various industries, government, and public sectors as well as intelligent manufacturing. The emergence of a novel and innovative four-dimensional printing (4D Printing) had allowed us to use AM technology and programmable materials to transform digital information in the virtual world into physical objects in the physical world to provide innovation and unprecedented functionality [2]. 4D printing was termed and defined by the Tibbits’ group at Massachusetts Institute of Technology (MIT) in 2013 and ever since then, the relative materials, 3D printers, stimulus mechanism, rules for innovative design and applications have been widely investigated and studied [3]. 4D printing allowed an additional dimension to be added onto the three-dimensional object where the shape, property, or functionality can be changed as a function of time. Through the stimulus activation of light, humidity, pH, solvent, magnetism, electric response or tension/compression etc., the 3D printed static object can converted into another predicable structure [4]. According to Momeni et al., 4D printing includes five elements, namely the 3D printing facility, stimulus-responsive materials/smart materials, stimulus, mathematical modeling, and interaction mechanism [5]. This concept implied that materials with various swelling ratios or thermal expansion coefficients could be printed via 3D printing facilities and further modified according to their shape-shifting behaviors [6]. In addition, 4D printing can also be defined as stimulus-responsive active smart materials that are mechanically deformed into other relevant shapes by using an external stimulus [5].

### 1.2. Shape-Shifting Behaviors

The main characteristic of 4D printing lies in the shape-shifting capabilities of a 3D printed static object. Shape variations in 4D printed objects can be induced by different external stimuli to cause programmed shrinkage, expansion or folding of the printed objects [7,8]. The main difference between 3D and 4D printing lies in the shape changing material (SCM) used, which is the advanced material that exhibits specific changes in response to external conditions. SCM is termed as such due to its shape-shifting behavior, which is defined as the capability to reversibly change its shape and/or dimensions in response to certain stimuli [7].

Another interesting factor of shape memory materials is the ability of the materials to retain a temporary memory of its shaped structure after the stimulus is applied to the SCMs [9,10]. This temporary shape can be held even after the stimulus is removed. By providing subsequent external stimulus, SCM could revert back to its original shape again. The polymeric material system of shape memory materials for 4D printing will be addressed in this article in order to explore its potential applications in medical fields [11].

### 1.3. Stimuli: Thermal, Hydro, pH, and Photo-Active Polymers

Yang et al. discussed two types of mechanisms in 4D printing, which are thermo- and hydro-mechanics [12]. Momeni et al. also named eight mechanisms and focused mainly on the mechanism theories and its related studies [5]. The following section briefly describes the four stimuli, heat, water, pH and optical mechanics, and the deformation mechanism of their associated polymers [13]. Thermo-responsive polymers are polymers that exhibit changes of their entropy elasticity, from a strained configuration (temporary state) to a less-ordered configuration (memorized state) under temperature changes. At a temperature which is higher than the glass transition temperature (*T*_g_), the original orientations of the polymeric chains are altered and thus resulted in new local chain-chain interactions. This temporarily deformed shape can be fixed as the material reaches a temperature which is lower than *T*_g_, as long as the new chain-chain interactions remain stable. These interactions are usually strong enough to overcome elastic recoiling, thus temporarily retaining their newly formed shapes.

Hydro-active polymers meant that the composite polymer is composed of highly hydrophilic expandable elements and non-active rigid elements. Water or moisture are utilized as the external stimulus for this type of polymers to actively change their shape. Usually, this type of shape change could be reversed once the moisture element is removed or after the polymers are dried. Furthermore, through special design, the polymers could be programmed such that the shapes remained irreversible, even after the moisture is removed [14,15]. pH-active polymers could be stimulated by adjusting the local pH and they undergo a coil-globule transition during pH changes. This coil-globule transition meant the collapse of a polymer from an expanded state to a compacted globule state after immersion in solvents with pH variations. 3D-printed pH-responsive hydrogels can expand at a specific pH level and condense at certain pH level in an aqueous environment [16].

In addition, light-induced polymers indicate that the shape changes can be programmed and recovered by using photo stimuli. There needs to be two levels of light induction in order for photo-active polymers to change and retain their shapes [17]. As mentioned previously, a deformed polymer shape-changed by light irradiation is also able to temporarily retain its shape even after the external stimulus is removed. By applying certain wavelength of light, photo-sensitive cross-links are cleaved or formed, thus allowing polymers to retain or return to their intended shapes [18].

## 2. 4D Fabrication

### 2.1. From 3D to 4D Fabrication

**Figure 1:**
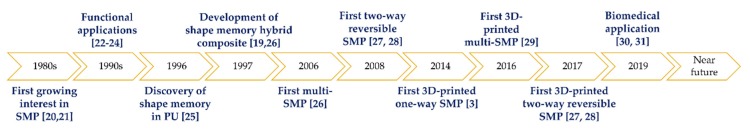
Progress and advancement of additive manufacturing techniques [34]. Copyright (2017) Elsevier.

The emergence of additive manufacturing (AM) techniques in the 1980s allowed scientists to explore and develop different types of shape memory polymers (SMP). Since then, SMP had garnered intense attention and continuous efforts from scientists (Figure 1) [19,20,21,22,23,24,25,26,27,28,29,30,31]. There were several AM fabrication tools such as fused deposition modeling, stereolithography, digital light processing, selective laser sintering/melting, binder jetting, and material jetting which allowed a wide range of materials and composites to be produced in complex shapes and 3D structures. Extensive studies on food, ceramics, thermosets, and thermoplastic composites using 3D printing technologies had already been well studied. However, 3D printing is still not widely applied in certain industries due to its slow speed in mass productions [19,20,21,22,23,24]. Interestingly, this drawback is considered as an advantage in precision medicine and customizable therapy because cases tend to vary from one patient to another. Therefore, 3D printing is generally well accepted and widely applied in biomedical or clinical applications [25,26,27,28,29,30]. Ever since THEN, scientists had attempted to map human organs, convert them into 3D virtual designs and are now attempting to find bio-comparable materials to fabricate vascularized human organs [31,32,33].

**Figure 2:**
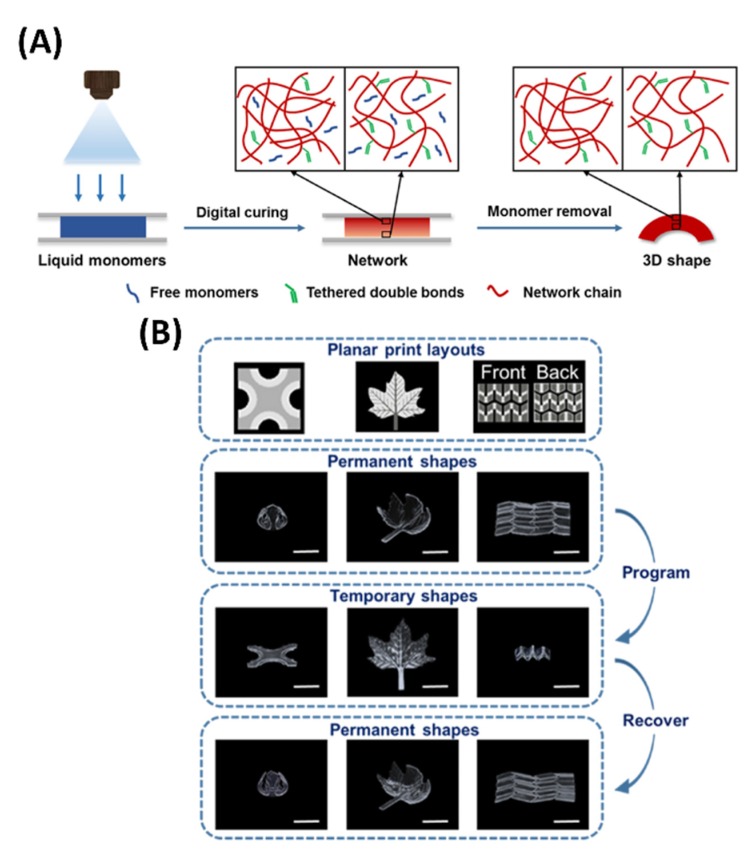
(**a**) Fabricate illustration of the digital shape memory polymers (SMP) process. (**b**) Digital fabrication of complex permanent shapes and proven of their shape memory behavior. In the planar print layouts, the black background represents no light exposure and the light and dark regions correspond to light exposure of 14 and 30 s, respectively [36]. Copyright (2019) American Chemical Society.

The merger of 3D printing technology and smart materials meant that we are able to produce 4D activated objects under external stimuli over time [35]. Smart materials have the ability to change shape with proper stimulus, and the discovery of smart materials gives rise and meaning to the terminology of 4D printing. Shape changing can be due to thermo-responsiveness, chemo-responsiveness, or used in combination to attain a structural reconfiguration between a temporary and a permanent shape. Thus, SMP materials could be engineered through 3D printing techniques into a ready-to-transform 4D printed object. Figure 2A depicted an illustration showing digital light from a commercial printer irradiating onto a printable solution sandwiched between two slides separated by a spacer. With these setups, it was reported that light could attenuate along the gradient or the difference in thickness thus allowing us to produce different curing conditions. Subsequent removal of the unreacted chemical agent would allow us have a polymer film and different structural shapes, while a difference in thickness allowed us to have in-built stress in the films. Release of stress from the films transformed the flat film into a 3D structure [36]. Figure 2B showed that the selection of planar light patterns allowed easy access to complex permanent shapes, which could be changed into temporary shapes and recovered upon reheating. This was due to the fact that certain SMP hydrogels were able to undergo certain structural arrangement according to the degree of swelling when exposed to certain stimuli [37]. In addition, Wu’s group reported a reversible shape change of ordinary and auxetic structures using 4D printing [38].

Since dynamic structural transformations depend on the initial printed shape, Mahadevan’s group in 2018 developed a visualization tool to convert a discrete geometry of filament bilayers into continuous plates with inhomogeneous growth patterns and thicknesses [39]. The evolution of the shapes and corresponding residual stresses and strains within the printed object could then be analyzed before experiments [40]. The main difference between 3D and 4D printings is that 4D printing not only makes a printed structure alive but also equips the structure with required functionality [41].

### 2.2. 4D Fabrication by Composites and Multimaterials

Tibbits et al. first introduced the concept of 4D fabrication into the architectural field in 2013. They designed constructs with rigid plastic materials and hydrophilic rubber using the Connex Objet500 printer. With the combination of multi-hydrogel and structure design, Gladman et al. synthesized a novel composite with nano-fibrillated cellulose, nano-clay and photoinitiator that could be fabricated into a programmable bi-layer structure using 4D-printing [42]. The printed scaffolds with different stiffness enabled the 3D-printed scaffold to have localized, anisotropic swelling behaviors. Therefore, precise control of the curvature in bilayer hydrogel composite structures can be harnessed to achieve complex 3D-to-4D shape transformation. In addition, there were various chemical- and temperature-responsive hydrogel-based materials that can be also used to perform 4D printing due to its non-uniform swelling-induced eigenstrains by modulating both the cross-linking density and location of the responsive gels [43,44].

**Figure 3:**
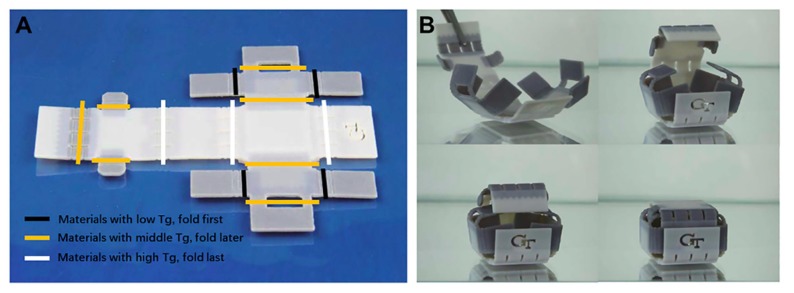
(**a**) The image of 3D printed self-folding box that combined different materials at different hinges. (**b**) The schematic diagram of a self-locking with a different external environment upon heating [56]. Copyright (2015) Nature Publishing Group.

A variety of functional chemical compounds were added together to achieve smart multi-materials. Functional groups can be activated using a temporary or a permanent shape. A very interesting example is the art of origami where a small piece of two-dimensional flat paper consisting of several photocurable compounds is folded into a large 3D object [45,46]. It is a bottom up design to allow self-folding or self-unfolding of two-dimensional sheets [47,48]. Actuators for soft robotics [49,50], hybrid soft electronics [51], a gripper system [52,53], and elastic bilayers were all fabricated using similar sophisticated multi-materials with 4D printing [54]. Polyurethane-based SMPs and its carbon nano-tube composites (SMP/CNT) are one of the multi-materials 4D building blocks that had been widely studied and developed. In the measurements of voltage variation, results revealed that the recovery times of the SMP/CNT samples were significantly reduced as the stimulus temperatures goes beyond the *T*_g_ of the specimens [55]. Since 3D printing hinges on using different *T*_g_ materials, adaptive designs capable of self-folding were created that respond rapidly to a heat stimulus and controlled shape-changing in succession (Figure 3) [56].

## 3. Materials: Shape Memory Polymers

### 3.1. Active Expandable Polymers

**Figure 4:**
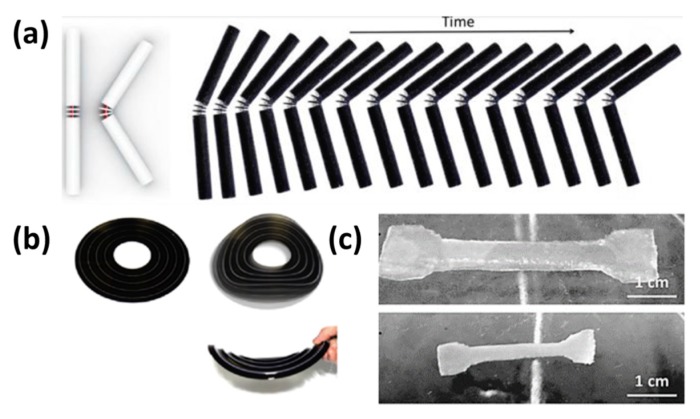
(**a**) The rendered image of the folded element, containing the structure of bars and disks. The center disk acts as a stop. The final folding angle can be set by adjusting the distance between the stops (left). The video frame of the fabricated element folding in water over time (right) [15]. Copyright (2016) Springer Nature. (**b**) The images of transformation from a flat 4D printed structure to a curve-crease origami structure [57]. (**c**) The images of the 3D printed hydrogel tensile specimen swollen in water at a 20 °C (top) and 60 °C (bottom) [58]. Copyright (2015) Wiley-VCH Verlag GmbH.

In 2014, Raviv’s group successfully used a combination of rigid plastics and hydrophilic UV curable polymers to fabricate a functional multi-material object using the Stratasys Connex 350 printer [15]. Because of the expandable property of these hydrophilic materials, the polymer can become a hydrogel-like expandable material that can grow and increase in size up to 200% when encountered with an aqueous environment. These hydrophilic acrylate monomers can be further cross-linked or transformed even after the process of polymerization thus allowing the material to swell whenever it comes into contact with an aqueous environment. These special design with center disks stoppers made various final folding angles possible (Figure 4a). However, there are some limitations in this composite 4D materials. This material is fragile and has a linear stretching limitation, thus severely limiting its application. Researchers still have a long way to go in order to solve the above-mentioned issues and enhance its applicability in the future. Moreover, there is a need to evaluate the physical properties of these water triggered materials for its future applications and to simulate and study its potential behavior at the molecular level.

The folding method was utilized with a simple but very useful logic by partially printing a material with rigidity and expandable property. A shape-shifting demonstration of Crambin protein from a 1D strand to a 3D structure was done by altering the stress mismatches between the active and rigid elements and its varied swelling properties. Even though these materials can be used in various applications due to its swelling capabilities, there is still a huge challenge to solve regarding its reversibility in multi-cycle applications. Moreover, the rings of the transformable flat sheet (Figure 4b) were manufactured with expandable materials separated by rigid materials so as to allow it to be shaped like a valley fold thus yielding scientifically mathematical sinusoidal surfaces under water [57]. Additionally, Bakarich’s group reported that such a specific design had a 3D-to-3D linear contraction and expansion behavior due to the linear free shrinkage or swelling of the thermo-responsive 10% (w/v) *N*-isopropylacrylamide, alginate/poly(*N*-isopropylacrylamide) (PNIPAAm) ionic covalent entanglement (ICE) hydrogels in the cold or hot water [58].

### 3.2. Additive Properties of Shape Memory Polymers

Multi-material SMPs had many useful characteristics such as controlled biodegradability, improved biocompatibility, conductivity, and thermal properties. Synthetic polymers used in biomedical applications, such as PCL and polylactic acid (PLA) based SMPs, are biodegradable SMPs derived from aliphatic polyesters which were found to induce little or no immunological reactions when compared with natural extracellular matrix (ECM) proteins [59]. In tissue engineering, the polymeric scaffolds fabricated from biocompatible and biodegradable materials have shown great ability in enhancing cell proliferation and new tissue formation [60]. PCL is a semi-crystalline polymer which can be synthesized by ring-opening polymerization of caprolactone using (SnO_2_) as the catalyst [61]. Polylactic acid (PLA) and its copolymers, such as polylactide-co-glycolide (PLGA), PLGA–poly(ethylene glycol) (PEG) are polymers with controllable biodegradability and good biocompatibility [62,63]. Poly(lactide-co-glycolide) (PLGA) is an important biodegradable polymer that has been widely used in the medical field [64]. These available materials and their composites can be used as SMPs, thus increasing the potential of multi-materials in clinical applications. In addition, numerous varied shaped memory nano-composites were developed to enhance electrical response and conductivity of SMPs. There was a good technique employed to make electro-active nano-composites by using a 3D conductive CNT network. The collective responses from the networks altered the electrical properties of the SMPs, thus resulting in a fast response timings with low voltages [65]. It was also noted that CNT coated SMP sheets resulted in a fast response timings at reduced voltages [66,67].

### 3.3. Multi-Structures

**Figure 5:**
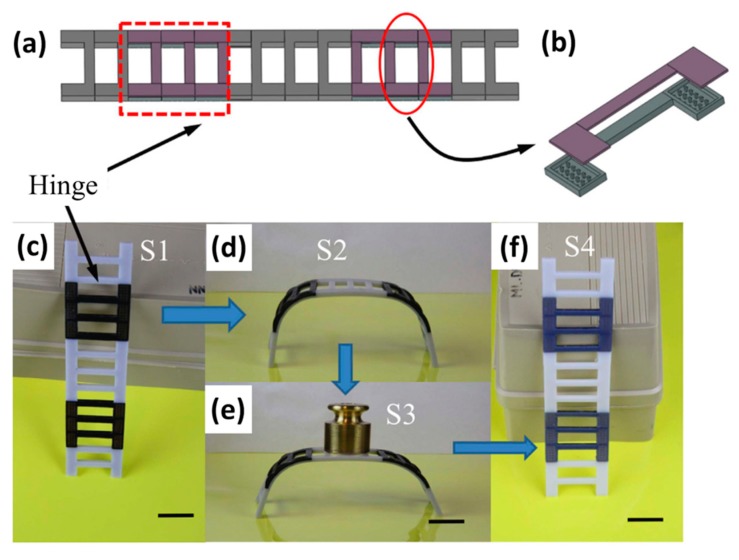
(**a**) The design of the ladder with two hinges. (**b**) Each hinge contains a reversible component. (**c**) The printed ladder. (**d**) After swelling for 10 h in low temperature water, it was bent into a bench shape, then heated to 75 °C, and then cooled to room temperature. (**e**) The bench is hard and can withstand a load of 50 g. (**f**) When the bench is placed in high temperature water (75 °C), the curved shape is restored. The scale bars represent 20 mm [70]. Copyright (2016) Springer Nature.

SMPs with special designs can be altered into different structures and several novel and interesting structures were described below. Duigou et al. used FDM to print a natural fiber bio-composite (hygromorph) with a two-layer microstructure. In addition, the double layer bio-composite can be driven by a water gradient. Due to the different porosity of the microstructure, variations in printed thickness can control stiffness and expansion capacity of the bio-composite. However, even though increment in filament thickness improves porosity, it was reported to otherwise reduce material-material cohesion, thus resulting in reduced tensile stress and increased water absorption [68]. Yang et al. used thermoplastic shape memory polyurethane (SMPU) as raw materials and prepared them into filamentary materials for FDM printing into shapes of flowers and aircrafts with complexed 3D structures [69]. In addition, Mao et al. used a composite material as a hinge to successfully design and print a reversible ladder (Figure 5) [70]. This design uses the hydrogel swelling property as the driving force for the shape change as the ladder was reported to be able to bear 50 g of pressure at room temperature. Another interesting design by Senatov’s group used PLA-based materials to achieve porous 4D thermoplastic hydroxyapatite (HA)/PLA scaffolds with shaped memories [71]. Moreover, the commercially available garments fabricated with SMPs can be used for maintaining stable body temperature via restriction of heat loss when the body temperature is below *T*_g_ of the SMPs. Crespy et al. incorporated the thermal-responsive hydrogels in textiles in order to obtain a fine balance between heat loss/gain and moisture [72]. Additionally, Yu et al. implemented a 4D printing epoxy-acrylate hybrid photosensitive polymer using optical SLA technology [73].

## 4. SMPs in Biomedical Applications

### 4.1. Drug Delivery Applications

Biomaterials for medical applications have stringent requirements and regulations. However, such desired characteristics can be overcome by selecting an appropriate material and processes [74]. Advanced polymers with various functions had been developed such as having bio-degradable shape-memory effects or combination of controlled drug release according to its biodegradability [75]. An example of such a combination is a minimally invasive implantable device commonly used for controlled drug release, for anti-inflammatory purposes or for initiating regeneration processes [76,77,78]. Pharmaceutical products based on shape-memory polymer can have controlled drug release and can also function as bio-functional implants. It was shown that a small change in the molecular structures of the polymers can cause vast differences in macroscopic properties, thus allowing us to customize a product suited for one’s needs [79].

**Figure 6:**
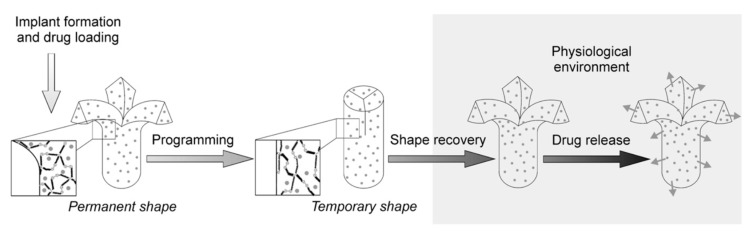
The concept of shape recovery, programming, and drug release of drug loaded SMP devices [80]. Copyright (2009) Elsevier.

Controlled drug release pharmaceutical material is the newest concept of functionalities of SMP and this concept is depicted in Figure 6 [80]. It is important to note that these different capabilities can be easily customized and/or tailored according to one’s needs, which makes such techniques attractive for most biomedical industries [80]. Even though it had several disadvantages which includes solubility, it was reported that such a drug loaded device requires much lower drug doses than oral administration. Furthermore, such drug delivery devices allowed us to facilitate and control the duration of drug release such as through a quick release or delayed release of drugs.

Therefore, a novel chemically cross-linked polymeric networks of branched oligo-(*ε*-caprolactone) biodegradable SMP was since developed to attempt to overcome the above-mentioned problems. This particular SMP exhibited a strain recovery rate of approximately 100% and it had a distinctly pronounced temperature-sensitive shape recovery. SMP loaded with theophylline (10 and 20 wt%) was further prepared by crosslinking branched oligo(*ε*-caprolactone) with hexamethylene diisocyanate in the presence of a specific amount of theophylline [81]. The 10 wt% material loaded with theophylline was flexible and soft enough to undergo shape transformation, and exhibited high strain recovery (99%) and strain fixability (98%). The theophylline could achieve a slow sustained release over one month and there was no initial burst-release in phosphate buffered saline (PBS) at 37 °C. These results showed that the use of biodegradable SMP is relevant and important for the development of drug delivery systems [81].

Furthermore, Malachowski et al. fabricated thermo-responsive multi-fingered grippers that were meant for controlled drug release in the gastrointestinal tracts. In their study, these grippers were made using traditional photolithography methods with rigid poly(propylene fumarate) sections and stimuli-responsive poly(*N*-isopropylacrylamide-*co*-acrylic acid) hinges. Once these grippers were inserted into the human body and were exposed to body temperature, they were shown to be able to grip onto our gastrointestinal tracts and allow sustained release of different types of drugs [82]. It had since then been shown that it was possible to achieve targeted drug delivery to specific intended locations in our body and the evolution and development of 4D printing had made it even more possible. Due to the possibility of having different types of stimuli, 4D printed devices can now be used to pack pharmaceutical drugs and to release them into the specific environment when that environment provides the device with the appropriate stimulus [83]. The first few related studies were done by Azam et al. during the early 2010s. Azam et al. fabricated containers using traditional photolithography made up of SU-8 photo-resistant panels and biodegradable PCL hinges that are thermo-responsive [84]. Once the containers reached their intended range of temperatures, the PCL hinge will close and thus allow the release of medications. Furthermore, in the study, the SU-8 panels had different porosities with different arrangement and sizes to study the rate of release of the containers.

### 4.2. Tissue Engineering Applications

Tissue engineering is designed to replace damaged tissues or to maintain and improve their functions. Tissue engineering is the use of a combination of cultured cells and scaffolds to form tissues which can then be implanted into the human body. Because of the unique properties of SMPs, it can be used as a scaffold to integrate newly formed tissues into living bones [85]. Furthermore, SMPs can be delivered to the injury site via minimally invasive technologies rather than the traditional surgical openings [86,87]. Once activated, either by thermal stimulation or moisture stimulation [88], it will have a configuration change, where the precise geometry and shape can be decided prior to fabrication [89]. There was a recent trial involving SMPs and intestinal disorders. Intestinal failures (IF) is termed when one is unable to maintain his/her body weight or sustain normal growth and was found to electrolyte homeostasis and enteral nutrition imbalance caused by shortened intestinal length [64]. Surgeons had designed several surgical interventions to attempt to solve IF such as colonic transposition, intestinal elongation and intestinal valve placement. Surgeons had also recommended tapering enteroplasty for short bowel syndrome (SBS)–IF patients to avoid intestinal dilation. Success rates vary and many complications may occur after surgery which includes anastomotic leakage, valve necrosis and obstruction [90].

**Figure 7:**
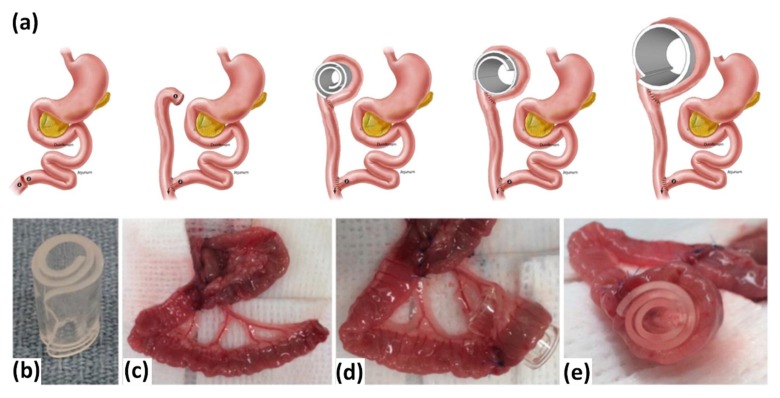
(**a**) The schematic for experimental animals (over 7 days); creation of Roux-en-Y limb, placement of cylindrical coil and radial expansion. (**b**) The image of radial expanding shape memory polymer device and (**c**) the end of Roux limb. (**d**) and (**e**) The device placed and wrapped by Roux limb [94]. Copyright (2015) Elsevier.

One promising therapy for SBS is to induce intestinal tissue regeneration or inducing distraction enter genesis by applying mechanical stretch. The applied tension induces growth through several known molecular mechanical transduction pathways which includes stimulating mesenteric neovascularization [91,92]. In previous studies, self-expanding polymer devices had been fabricated and tested in small animal models [93]. The results showed that mechanical stretch or elongation provided by the SMPs were able to promote growth of intestinal tissues that were proven to have similar characteristics as native tissues (Figure 7) [94]. The acrylic based network with hydrophobic and hydrophilic monomers were able to control the rate of activation and swelling. 65 °C was used to sterilize to SMP as this temperature was close to the Tg of the polymers so as to prevent early activation. A thin rectangular sheet was then placed into 80 °C water and wounded onto a mandrel with a diameter of 8.5 mm and height of 15 mm to allow shape formation. After which the sheet was tied down with a tie to prevent it from loosening during cooling and sterilization. After implantation into a rat model, it was reported that the intestine grew to twice its original length and there were no significant differences between the muscle thickness of the original tissue and the newly grown tissue. This study showed that such a self-generation method could be used for SBS-IF patients instead of the traditional surgical and intrusive methods [94].

**Figure 8:**
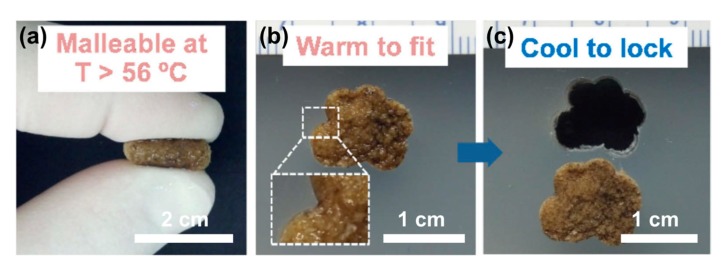
The images of self-fitting behavior of a polydopamine-coated PCL SMP scaffold. When heated above the Tm of the PCL, the cylindrical support softens, making it mechanically suitable for model irregularities. After cooling, the new temporary shape remains even after the bracket is removed from the defect [95]. Copyright (2014) Elsevier.

Recently, a novel SMP was also developed to treat cranio-maxillofacial defects (Figure 8) [95,96]. A PCL diacrylate was briefly treated with warm saline, UV polymerized, then treated with a solvent-cast particulate leaching method to create porous scaffolds. The “self-fitting” behavior of the SMP into irregular defects allowed us to create customizable shapes and structures suited to the patient’s needs [97].

**Figure 9:**
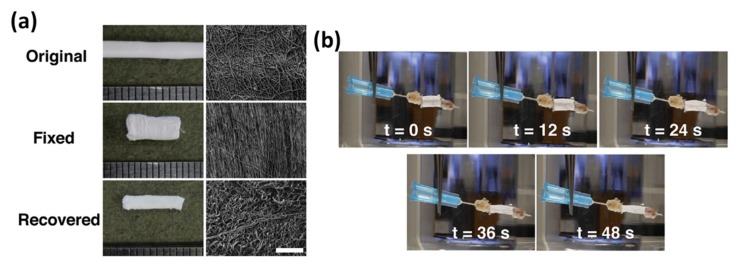
(**a**) The images of SMP sleeve in different conditions. (**b**) SMP sleeve contracting around a femur with a 4-mm segmental defect filled with allograft. Recovery preformed within 50 s with heated saline at 45 °C [98]. Copyright (2016) Elsevier.

In 2015, Henderson’s group developed an expandable porous SMP as a graft or sleeve and implanted them into established rat models to fill complex bone defects (Figure 9) [98]. They used a modified porogen-leaching method to produce the porous SMPs grafts which were programmed to deform at room temperature to prevent low Tg and early shape changes. A thermoplastic polyurethane was electrospun onto the SMP to stabilize it. The sleeve was spread over a tapered cone from a length of 9 mm and 1.5 mm and with an inner diameter of 3.5 mm at 65 °C. It was then cooled to 22 °C for 1 min and subsequently removed from the water at room temperature. In a 12-week study, the results showed that the SMP graft and SMP sleeve were able to integrate well with natural bone. The graft or sleeve also displayed rapid reversal to original shape when placed into heated saline of 45 °C (Figure 9). Furthermore, an important aspect of stimuli responsive polymers lies in its ability to reverse the response, thus meaning that the material is able to restore to its normal shapes after removal of the stimuli. Song et al. mentioned an example found in nature that critically exhibited such a reversal. The scales of pine cones were found to react to the level of humidity in the environment. The scales contract once humidity rises and expand when the humidity is low and thus allowing scattering of their seeds [99]. To allow reversability, researchers had attempted to combine two polymers or ceramics with contrasting features to allow shape reversal [71]. In their study, it was shown that a combination of PLA with 15% hydroxyapatite was able to achieve 98% of shape reversal when the stimulus was removed.

Similarly, it is important for us to explore for polymers that are responsive to different stimuli so that it is possible to fabricate for different applications and purposes. Currently, studies had been reported regarding responsiveness to physical stimuli such as temperature, photo-responsive, magneto-responsive and also to chemical stimuli such as pH and humidity. It is important to consider the stimulating conditions of the specific environment so that we are able to fabricate constructs that suit our environment. Of which, temperature is the most commonly studied factor for 4D printing in tissue engineering. A recent review by Tamay et al. had reviewed and compiled the different factors and their relevant studies [100].

### 4.3. Wound-Dressing Products

Wound healing or lesion healing using dressing product is often an elaborate procedure involving an integrated physiological responses which includes hemostasis and blood clotting; inflammatory processes to clear unwanted debris; regeneration of connective tissue cells; matrix contraction and finally epithelization [101]. Wound dressings can be categorized into conventional biological skin substitute or artificial dressings [102]. However, conventional dressing materials often fail to allow humidity and moisture to pass through, thus making the wound dry and prone to infections [103]. Biological skin substitute dressings are also termed as “auto-grafting”, thus another major issue depends on whether the patient has available resources for skin grafting [104]. Therefore, researchers had fabricated advanced synthetic dressing’s material composition developed from polymeric membrane materials to cover for the shortfalls of auto-grafting. Dressing materials or natural-polymer must be bio-compatible, bio-degradable, non-toxic, and non-allergenic.

In this case, silk fibroin dressings have excellent characteristics which includes delayed degradation, non-immunogenic, high water absorption capacity, great air permeability and is also economically cheap. Furthermore, silk fibroin is an elastic polymer with outstanding mechanical properties which includes tensile strength 0.5 GPa, breaking elongation of 15% and elasticity of <35% [105]. Dressings based on hydrogel material were also in high demand due to their excellent characteristics. Currently, we are able to see higher quantities of hydrogel dressing in the market as compared to conventional gauze dressings [106,107]. An advanced wound dressing made from bacterial cellulose/acrylic acid hydrogel has recently emerged in the market as a partial-thickness burn wound dressing. Dressings made up of bio-materials were found to be more superior when compared to conventional dressings as they were able to mimic native skin tissues better. Therefore, a novel idea of seeding dermal fibroblasts and human epidermal keratinocytes onto bio-material dressings were formed to treat full-thickness skin lesions [108]. In this aspect, chitosan was reported to have good wound healing and antibacterial properties and is currently widely used in the production of wound dressing products to help rebuild skin structures. The concept of utilizing chitosan is not new. In the past, chitosan-managed cotton fabrics and alginate filaments have been used for fabrication of wound dressings [109]. Several natural polymer-based hydrogel products have also been promoted for wound dressing applications [110]. Therapeutic drug molecules can be incorporated into such intelligent wound dressings and can be activated in response to pH variations/temperature changes at desired tissue site to allow wound remodeling and regeneration.

### 4.4. Skin-Care Products

A special feature of SMPs in skin-care products lies in its ability to have shape reversal properties which allowed it to return to its original shape and structure after external stimulation [111]. Recently, several shape-memory materials of skin-care products had already been used in textiles, and studies had shown that they might have great potential for future cosmetics applications. Cosmetotextiles are textiles that have skin-care characteristics and they contain an active substance that is able to release into human body according to our needs. The stimuli-responsive skin-care products was prepared by using an aroma, various kinds of skin softeners, phase-change materials which helps in thermo-regulation of the body, as well as chemical or physical agents that has antimicrobial properties. Together, they function as a transport system to deliver pharmaceutical substances into our targets [112]. In the future, SMPs in skin care applications will be expected to be made in accordance to one’s skin properties and be able to be customizable to one’s need in order to restore our skin beauty. As such, biotechnology sectors are investing heavily in fabricating SMPs for skin care applications with highly bionic characteristics and yet at the same time, to be manageable at the cellular level [113].

## 5. Future Prospects

4D printing is an art that combines science and engineering techniques and has attracted a lot of interests due to its unique ability to have structural or functional transformations over time in response to external stimuli. The scientific aspects of 4D printing involve the development of basic research in mathematical modeling and new smart materials. As technology advances, printing methods, software programs, new materials and machines are constantly being developed and improved. 4D printing arises as a new technology that utilizes smart materials to design structures not only with the ability to transform in response to external stimuli, but are also predictable and can be easily monitored. This allows smart printing to be used in a variety of fields by monitoring the specific shape changes in the designed structures. The significant increase in cellular metabolism as well as cell proliferation on the materials are the perfect indications of having good cytocompatibility, making them good candidates for tissue engineering. In addition, many shape memory nano-composites are being developed to upgrade the electrical response and conductivity in neural cells.

Additive manufacturing is still a growing industry and the continuous exploration of new materials has led to significant improvements in the field of 4D printing. New technology provides feasible methods to fabricate compact deployable structures. However, new smart materials for 4D printing will be the key factor for its advanced applications not merely for medical but also for commercially available products in the future. In conjunction with the development of new smart materials and additive manufacturing technologies, potential applications for the 4D printing process can be maximized in the future, and 4D printing is expected to be of great benefit in the future.
